# Water-Deficit Tolerance in Sweet Potato [*Ipomoea batatas* (L.) Lam.] by Foliar Application of Paclobutrazol: Role of Soluble Sugar and Free Proline

**DOI:** 10.3389/fpls.2017.01400

**Published:** 2017-08-08

**Authors:** Suravoot Yooyongwech, Thapanee Samphumphuang, Rujira Tisarum, Cattarin Theerawitaya, Suriyan Cha-um

**Affiliations:** ^1^Division of Agricultural Science, Mahidol University Kanchanaburi, Thailand; ^2^National Center for Genetic Engineering and Biotechnology (BIOTEC), National Science and Technology Development Agency (NSTDA) Pathum Thani, Thailand

**Keywords:** sweet potato, paclobutrazol, water deficit, pigment, proline, total soluble sugar

## Abstract

The objective of this study was to elevate water deficit tolerance by improving soluble sugar and free proline accumulation, photosynthetic pigment stabilization, photosynthetic abilities, growth performance and storage root yield in sweet potato cv. ‘Tainung 57’ using a foliar application of paclobutrazol (PBZ). The experiment followed a Completely Randomized Block Design with four concentrations of PBZ: 0 (control), 17, 34, and 51 μM before exposure to 47.5% (well irrigation), 32.3% (mild water deficit) or 17.5% (severe water deficit) soil water content. A sweet potato cultivar, ‘Japanese Yellow’, with water deficit tolerance attributes was the positive check in this study. Total soluble sugar content (sucrose, glucose, and fructose) increased by 3.96-folds in ‘Tainung 57’ plants treated with 34 μM PBZ grown under 32.3% soil water content (SWC) compared to the untreated plants, adjusting osmotic potential in the leaves and controlling stomatal closure (represented by stomatal conductance and transpiration rate). In addition, under the same treatment, free proline content (2.15 μmol g^-1^ FW) increased by 3.84-folds when exposed to 17.5% SWC. PBZ had an improved effect on leaf size, vine length, photosynthetic pigment stability, chlorophyll fluorescence, and net photosynthetic rate; hence, delaying wilting symptoms and maintaining storage root yield (26.93 g plant^-1^) at the harvesting stage. A positive relationship between photon yield of PSII (Φ_PSII_) and net photosynthetic rate was demonstrated (*r*^2^ = 0.73). The study concludes that soluble sugar and free proline enrichment in PBZ-pretreated plants may play a critical role as major osmoprotectant to control leaf osmotic potential and stomatal closure when plants were subjected to low soil water content, therefore, maintaining the physiological and morphological characters as well as storage root yield.

## Introduction

Sweet potato is classified as a candidate rain-fed crop for the semi-arid and arid regions (Gomes and Carr, 2001, 2003; Gomes et al., 2005). However, climate change has resulted in drought or water shortage or water deficit (WD) conditions (Rockstrom, 2003; Blum, 2005; Neumann, 2008; Falkenmark, 2013). Under these conditions, crop water use efficiency, crop water content are directly influenced, leading to low net photosynthetic rate, growth reduction, and storage root yield loss (van Heeden and Laurie, 2008; Gajanayake et al., 2014; Yooyongwech et al., 2016). Biochemical, physiological, and morphological performances of sweet potato plants under WD conditions have been well assessed using multivariate cluster analysis and ‘Japanese Yellow’ and ‘PROC 65-3’ have been identified as WD tolerance cultivars (Yooyongwech et al., 2013). Soluble sugar enrichment and free proline accumulation at the cellular level play a key role in osmotic adjustment, which promotes drought defense mechanisms (Wang et al., 2000; Yooyongwech et al., 2016).

Triazole compounds such as paclobutrazol (PBZ) (Sankar et al., 2007), triadimefon (Manivannan et al., 2008), hexaconazole (Chehelpar et al., 2016), and uniconazole (Zhang et al., 2007) promote osmoregulatory effects on plants. Paclobutrazol [(2RS, 3RS)-1-4(-chlorophenyl)-4, dimethyl-2-1,2,4-triszol-1-yl-penten-3-ol] is a known gibberellic acid inhibitor (anti-GA) and has been applied as plant growth retardant. In sweet potato, flood tolerance (cv. ‘Taoyuan 2’; 5 days flooding) and chilling tolerance traits (cvs. ‘TN71’ and ‘TN65’; ambient temperature at 7°C for 5 days) have been reported using PBZ-pretreatment at 0.5 mg plant^-1^ and 300 μg plant^-1^, respectively. Under flooding and chilling stress, the regulation of antioxidative system, i.e., enzymatic and non-enzymatic defense mechanisms, in PBZ-pretreated plants were reported (Lin et al., 2006a,b). PBZ also induces drought tolerance in *Stevia rebaudiana* (Hajihashemi and Ehsanpour, 2013, 2014), *Aesculus hippocastanum* (Percival and Noviss, 2008), and *Arachis hypogaea* (Sankar et al., 2007). Moreover, the possible hypotheses on drought tolerance regulation by PBZ have been proposed which state that it maintains the endogenous cytokinin levels (zeatin and zeatin riboside) and stabilize leaf water potential (Zhu et al., 2004) causing increased leaf and epidermal thickness (Sankar et al., 2013). Alternatively, regulation of free proline and glycine betaine as major osmoprotectants (Hajihashemi and Ehsanpour, 2013) and promotion of enzymatic and non-enzymatic antioxidant activities, reduce the toxicity derived from drought stress (Sankar et al., 2007; Hajihashemi and Ehsanpour, 2014; Jungklang et al., 2016). However, knowledge on PBZ-regulated sugar and free proline enrichment in sweet potato grown under WD condition is limited. The aim of this investigation was to: (a) improve the WD tolerant abilities in sweet potato subjected to varying degrees of water stress using foliar PBZ concentrations, and (b) evaluate biochemical, physiological, and morphological characteristics and storage root traits in each treatment.

## Materials and Methods

### Plant Materials, PBZ-Pretreatment, and Water Deficit Treatments

Two sweet potato genotypes, namely ‘Japanese Yellow’ (WD tolerance; positive check) and ‘Tainung 57’ (WD sensitive) obtained from Agricultural Extension Group, Phichit province, Thailand, were used as master stock materials (Yooyongwech et al., 2013). Single vine cuttings (15 ± 1 cm in length) without leaf blades were propagated and planted into plastic pots (∅ = 20 cm) containing 2 kg garden soil (EC = 2.687 dS m^-1^; pH = 5.5; organic matter = 10.36%; total nitrogen = 0.17%; total phosphorus = 0.07%; and total potassium = 1.19%). The cuttings planted in the pot culture were incubated in a greenhouse under 500-1,000 μmol m^-2^ s^-1^ photosynthetic photon flux density with a 10 h d^-1^ photoperiod, 28 ± 2°C ambient temperature and 80 ± 5% relative humidity, for 4 weeks. The ‘Tainung 57’ plants were treated with exogenous foliar applications of PBZ at 0, 17, 34, and 51 μM (25 mL plant^-1^). Each treatment consisted of well-watered (WW; 47.5% soil water content), mild water deficit (MWD; 32.3% soil water content, by withholding water for 7 days), and severe water deficit (SWD; 17.5% soil water content, by withholding water for 14 days) conditions (Supplementary Figure S1). Growth characteristics (vine length and number of leaves), soluble sugar, free proline content, leaf osmotic potential, photosynthetic pigments, chlorophyll fluorescence, net photosynthetic rate (*P*_n_), stomatal conductance (*g*_s_), and transpiration rate (*E*) in each treatment was measured. In addition, number of storage roots per plant, storage root yield, vine fresh weight, root fresh weight, vine dry weight, and root dry weight in the harvesting period (150 days after cutting) were recorded.

### Biochemical Analysis

Sucrose, glucose, and fructose content in the second fully expanded leaf from shoot tip were measured following the method of Karkacier et al. (2003). In brief, sweet potato leaves were collected and freeze-dried using a freeze-dryer. Fifty-milligram sample was ground in a mortar with liquid nitrogen. Following this, 1 mL of nanopure water was added and centrifuged at 12,000 rpm for 15 min. The supernatant was collected and filtered through a 0.45 μm membrane filter (VertiPure^TM^, Vertical^®^). Twenty microliters of the filtrate was injected into Waters HPLC equipped with a MetaCarb 87C column and a guard column. Deionized water was used as the mobile phase at a flow rate of 0.5 mL min^-1^. The online detection was performed using a Waters 410 differential refractometer detector and the data was analyzed by Empower^®^ software. Sucrose, glucose, and fructose (Fluka, United States) were used as the standards.

Free proline in the second fully expanded leaf from shoot tip was extracted and analyzed according to the method of Bates et al. (1973). In brief, 50 mg of fresh material was ground with liquid nitrogen in a mortar. The homogenate powder was mixed with 1 mL aqueous sulfosalicylic acid (3%, w/v) and filtered through Whatman #1 filter paper (Whatman, England). The extracted solution was reacted with an equal volume of glacial acetic acid and ninhydrin reagent (1.25 mg ninhydrin in 30 mL glacial acetic acid and 20 mL 6 M H_3_PO_4_) and incubated at 95°C for 1 h. The reaction was terminated by placing the container in an ice bath. The reaction mixture was mixed vigorously with 2 mL of toluene. After cooling to 25°C, the chromophore was measured at 520 nm by spectrophotometer (HACH DR/4000; Model 48000, HACH Company, Loveland, CO, United States) using L-proline as a calibration standard.

### Physiological Characters

Osmotic potential in the leaves of sweet potato was measured, according to Lanfermeijer et al. (1991). In brief, 100 mg of fresh leaf tissue was chopped into small pieces, transferred to 1.5 mL micro tube, and then crushed using a glass rod. The 20 μL of extracted solution was dropped directly onto a filter paper in an osmometer chamber (5520 Vapro^®^, Wescor, Logan, UT, United States) and subsequently the data were collected. The osmolarity (mmol kg^-1^) was converted to osmotic potential (MPa) using conversion factor of osmotic potential measurement.

Chlorophyll a (Chl *a*), chlorophyll b (Chl *b*), total chlorophyll (TC), and total carotenoid content in the second fully expanded leaf from shoot tip were analyzed according to the method of Shabala et al. (1998), whereas total carotenoid (C_x+c_) content was assayed following Lichtenthaler (1987) method. One hundred milligram leaf tissue was homogenized in glass vials using 10 mL of 99.5% acetone, and blended using a homogenizer. The glass vials were sealed with Parafilm^®^ to prevent evaporation, and then stored at 4°C for 48 h. Chl *a* and Chl *b* concentrations were measured at 662 and 644 nm, whereas C_x+c_ concentration measured at 470 nm using UV-VIS spectrophotometer against acetone (99.5%) as a blank.

Chlorophyll fluorescence emission was measured from the adaxial surface of the second fully expanded leaf from the shoot tip using a fluorescence monitoring system (model FMS 2; Hansatech Instruments Ltd., Norfolk, United Kingdom) in the pulse amplitude modulation mode (Loggini et al., 1999). A leaf, kept in dark for 30 min was initially exposed to the modulated measuring beam of far-red light (LED source) with typical peak at 735 nm. Original (*F*_0_) and maximum (*F*_m_) fluorescence yields were measured under weak modulated red light (<0.5 μmol m^-2^ s^-1^) with 1.6 s pulses of saturating light (>6.8 μmol m^-2^ s^-1^ PAR) and calculated using FMS software for Windows^®^. The variable fluorescence yield (*F*_v_) was calculated using the equation: *F*_v_ = *F*_m_ - *F*_0_. The ratio of variable to maximum fluorescence (*F*_v_/*F*_m_) was calculated as the maximum quantum yield of PSII photochemistry. The photon yield of PSII (Φ_PSII_) in the light was calculated as: Φ_PSII_ = (*F*_m_′ -*F*)/*F*_m_′ after 45 s of illumination, when steady state was achieved (Maxwell and Johnson, 2000).

Net photosynthetic rate (*P*_n_; μmol m^-2^ s^-1^), stomatal conductance (*g*_s_; mmol H_2_O m^-2^ s^-1^), and transpiration rate (*E*; mmol m^-2^ s^-1^) of the second fully expanded leaf from shoot tip were measured using a Portable Photosynthesis System with an Infra-red Gas Analyzer (Model LI 6400, LI-COR^®^ Inc., Lincoln, NE, United States). Transpiration rate was measured continuously by monitoring the content of air entering and exiting the IRGA headspace chamber, according to Cha-um et al. (2007). Flow rate of IRGA headspace chamber was set as 500 μmol s^-1^ with ambient CO_2_ concentration (350 ± 10 μmol CO_2_ mol^-1^) and 1,000 μmol m^-2^ s^-1^ photosynthetic photon flux density provided by 6400-02 B LED Red/Blue light source.

### Growth Performances

Vine length, leaf length, leaf width, number of leaves, storage root yield, vine fresh weight, root fresh weight, vine dry weight, and root dry weight of sweet potato were measured. Vine and roots were dried at 80°C in a hot-air oven for 2 days, and then incubated in desiccator before the measurement of dry weight. In addition, the leaf toxic symptoms, i.e., leaf wilting and leaf chlorosis, were observed (Supplementary Figure S2).

### Experiment Design and Statistical Analysis

The experiment was arranged as 4 × 2 factorial in a Completely Randomized Block Design (CRBD) with eight replicates (*n* = 8). Two-way analysis of variance (ANOVA) in each parameter was validated using SPSS software (Supplementary Tables S1–S3). The mean values obtained were compared using Tukey’s HSD and analyzed with SPSS software. Pearson correlation between Chl *a* content and *F*_v_/*F*_m_, TC and Φ_PSII_, Φ_PSII_ and *P*_n_, and *P*_n_ reduction and plant dry weight was analyzed.

## Results

### Yield Traits

**FIGURE 1:**
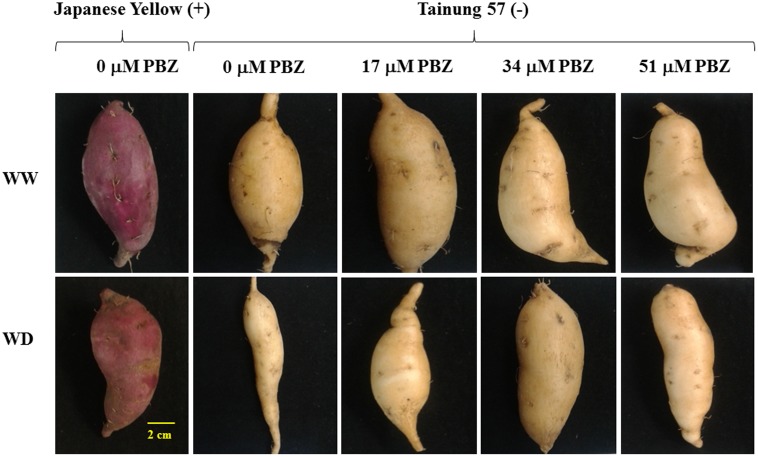
Storage root characteristics of sweet potato pretreated with paclobutrazol (PBZ) grown under well watering [WW; 47.5% soil water content (SWC)] or severe water deficit (SWD) (17.5% SWC; 14 days water withholding) subsequently recovery prior to storage root harvesting process (150 days after planting).

Morphological characteristics of the storage roots of sweet potato at the harvest stage are shown in **Figure 1**. There was no significant difference observed in the storage root yield of ‘Japanese Yellow’, the positive check, under WW and WD conditions (only 5.4% reduction). ‘Tainung 57’ plants grown under WW conditions and pretreated with PBZ had a higher storage root yield than the non-pretreated plants (**Figure 1**). In WD treatment, storage root yield was significantly improved especially in 37 μM PBZ pretreated plants (**Figure 1**) Storage root yield of untreated ‘Tainung 57’ was significantly decreased by 47.55% when exposed to 17.5% SWC (SWD) compared to WW conditions. However, when the plants were treated with 17, 34, and 51 μM of PBZ, the yield decreased up to 37.68, 35.77, and 34.95%, respectively (**Table 1**).

**Table 1:** Storage root yield, vine fresh weight, root fresh weight, vine dry weight, and root dry weight of sweet potato pretreated with paclobutrazol (PBZ) subsequently subjected to well watering [WW; 47.5% soil water content (SWC)] or severe water deficit (SWD; 17.5% SWC) and then recovered until storage root harvesting stage (150 days after cutting).

Treatment	Water deficit	Storage root yield (g)	Vine FW (g)	Root FW (g)	Vine DW (g)	Root DW (g)
**cv. ‘Japanese Yellow’ (+)**						
	WW	21.23ef	24.44e	3.26cd	3.60b	1.28ab
	WD	20.08ef	19.66e	2.40de	2.45c	0.71c
		(5.42%)	(19.52%)	(26.38%)	(31.94%)	(44.53%)
**cv. ‘Tainung 57’ (-)**						
0 μM PBZ	WW	31.46cd	58.50c	3.62cd	2.52c	1.39ab
	WD	16.50f	35.04d	1.25f	0.81d	0.46c
		(47.55%)	(40.10%)	(65.47%)	(67.86%)	(66.91%)
17 μM PBZ	WW	60.46a	86.98a	4.33bc	3.53bc	1.27ab
	WD	37.68bc	66.82bc	2.81de	2.41c	0.53c
		(37.68%)	(23.18%)	(35.10%)	(31.73%)	(58.27%)
34 μM PBZ	WW	41.93b	72.41b	4.86ab	6.13a	1.29ab
	WD	26.93de	58.19c	2.78de	3.42bc	0.67c
		(35.77%)	(19.64%)	(42.80%)	(44.21%)	(48.06%)
51 μM PBZ	WW	43.44b	77.51ab	5.96a	5.39a	1.78a
	WD	28.26cd	37.28d	2.52de	2.04c	0.99bc
		(34.95%)	(51.89%)	(57.72%)	(62.15%)	(44.38%)


*Different letters in each column show significant difference at *p* ≤ 0.01 by Tukey’s HSD. Figures in parentheses represent yield reduction percentage of water-deficit stressed plants compare to WW in each cultivar.*

Vine fresh weight, root fresh weight, vine dry weight, and root dry weight in PBZ-pretreated plants raised under WW conditions were greater than those in water stress. In WD stress conditions, vine fresh weight, root fresh weight, vine dry weight, and root dry weight in non-pretreated plants declined by 40.10, 65.47, 67.86, and 66.91%, respectively; whereas, these parameters improved with the 34 μM PBZ treatment (19.64, 42.80, 44.21, and 48.06%, respectively). In addition, under WD conditions the root fresh weight (35.10% reduction) and vine dry weight (31.73% reduction) in 17 μM PBZ-pretreated plants were maintained. In the positive check cv. ‘Japanese Yellow,’ the above traits in WD stressed plants showed higher performance than the ‘Tainung 57’ plants (**Table 1**).

### Morphological Characters

Leaf wilting and chlorosis were evidently observed in the older leaves when sweet potato plants were exposed to SWD, especially in non-treated plants (Supplementary Figure S2). Vine length was significantly reduced by PBZ treatment, whereas leaf length, leaf width, and number of leaves remained same. In WW conditions, plant height or vine length of sweet potato pretreated with 34 μM PBZ was reduced by 18.78% when compared with control. Under MWD conditions (32.3% SWC), vine length, leaf area, leaf width, and number of leaves of sweet potato cv. ‘Japanese Yellow’ were maintained (with only 6.96, 2.54, 4.25, and 6.04% reduction, respectively) with a lower reduction rate as compared to the cv. ‘Tainung 57’ (with 31.62, 10.01, 13.78, and 19.36% reduction, respectively). The degree of reduction in each parameter was amplified with the extent of water deficiency and genetic variations, especially in cv. ‘Tainung 57’ (**Table 2**). In PBZ-pretreated plants, overall growth performances under WD conditions were alleviated. Interestingly, vine length, leaf area, leaf width, and number of leaves in 34 μM PBZ pretreated plants of cv. ‘Tainung 57’ were maintained in both MWD (10.52, 3.09, 0.79, and 3.13% reduction) and SWD (23.02%, 13.00%, 20.10% and 34.92% reduction) (**Table 2**).

**Table 2:** Vine length, leaf length, leaf width, and number of leaves of sweet potato pretreated with PBZ subsequently subjected to WW (47.5% SWC) or mild water deficit (32.3% SWC) for 7 days or SWD (17.5% SWC) for 14 days.

Treatment	Water deficit	7 days				14 days			
			
		Vain length (cm)	Leaf length (cm)	Leaf width (cm)	Number of leaves	Vain length (cm)	Leaf length (cm)	Leaf width (cm)	Number of leaves
**cv. ‘Japanese Yellow’ (+)**									
	WW	35.21bc	8.85b	6.82c	14.9a	43.67bc	9.46bcd	8.70abc	19.25a
	WD	32.76c	8.73b	6.53c	14.0a	40.20c	9.22bcd	8.17bc	17.67ab
		(6.96%)	(2.54%)	(4.25%)	(6.04%)	(7.95%)	(1.36%)	(6.09%)	(8.21%)
**cv. ‘Tainung 57’ (-)**									
0 μM PBZ	WW	50.48a	10.49a	10.52a	12.4b	67.90a	10.98a	10.62a	18.33a
	WD	34.52bc	9.44ab	9.07ab	10.0c	33.38d	8.44cd	8.12bc	7.38c
		(31.62%)	(10.01%)	(13.78%)	(19.36%)	(50.84%)	(23.13%)	(23.54%)	(59.74%)
17 μM PBZ	WW	40.42b	10.40a	9.95ab	12.2b	59.95ab	10.60ab	10.15abc	16.50b
	WD	34.60bc	9.67ab	9.03ab	10.8bc	39.73c	8.75cd	7.99c	9.44c
		(14.40%)	(7.02%)	(9.25%)	(11.48%)	(33.73%)	(17.45%)	(21.28%)	(42.79%)
34 μM PBZ	WW	41.05b	9.71ab	8.87ab	12.8b	55.13bc	9.23bcd	10.05abc	13.83bc
	WD	36.73bc	9.40ab	8.80ab	12.4b	42.44c	8.03d	8.03bc	9.00c
		(10.52%)	(3.09%)	(0.79%)	(3.13%)	(23.02%)	(13.00%)	(20.10%)	(34.92%)
51 μM PBZ	WW	45.97ab	8.98b	8.89ab	12.1b	55.44bc	10.32abc	10.26ab	18.60a
	WD	33.68c	8.60b	8.44b	11.4bc	34.90d	8.04d	8.08bc	8.00c
		(26.74%)	(4.23%)	(5.06%)	(5.79%)	(37.05%)	(22.09%)	(21.25%)	(56.99%)


*Different letters in each column show significant difference at *p* ≤ 0.01 by Tukey’s HSD. Figures in parentheses represent growth reduction percentage of water-deficit stressed plants compare to WW in each cultivar.*

### Biochemical Changes, Osmotic Adjustment, and Stomatal Function

**FIGURE 2:**
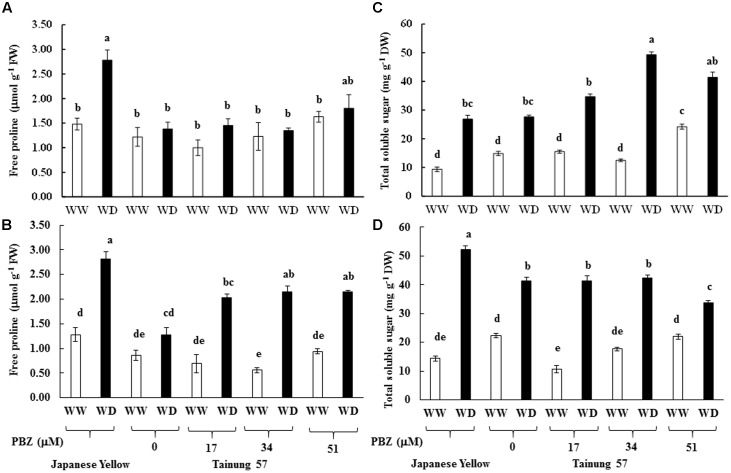
Free proline and total soluble sugar in leaf tissues of sweet potato pretreated with PBZ subsequently subjected to mild water deficit (32.3% SWC; 7 days water withholding) **(A,C)** or SWD (17.5% SWC; 14 days water withholding) **(B,D)**. Different letters in each bar show significant difference at *p* ≤ 0.01 by Tukey’s HSD. Data presented as mean ± SE.

Free proline in sweet potato cv. ‘Japanese Yellow’ grown under MWD was enriched by 1.87-fold (2.78 μmol g^-1^ FW) over the WW control. However, the proline levels of PBZ pretreated and untreated cv. ‘Tainung 57’ were not significantly different when exposure to MWD (**Figure 2A**). In SWD, free proline peaked (2.81 μmol g^-1^ FW) in the positive check cv. ‘Japanese Yellow’, while it was maintained in cv. ‘Tainung 57’ without PBZ treatment (**Figure 2B**). In addition, free proline content in PBZ-pretreated cv. ‘Tainung 57’ grown under SWD was significantly enriched by 2.94- (2.03 μmol g^-1^ FW), 3.84- (2.15 μmol g^-1^ FW), and 2.28-fold (2.14 μmol g^-1^ FW), when pretreated with 17, 34, and 51 μM PBZ, respectively (**Figure 2B**).

Total soluble sugar in MWD stressed plants increased by 2.87- (26.85 mg g^-1^ DW) and 1.85-folds (27.61 mg g^-1^ DW) over the control in cvs. ‘Japanese Yellow’ and ‘Tainung 57,’ respectively. The increase was most prominent at 34 μM PBZ where the total soluble sugar concentration was 49.33 mg g^-1^ DW (3.69-fold over control) (**Figure 2C**). Under SWD conditions, total soluble sugar was enriched in both the positive check cv. ‘Japanese Yellow’ (3.63-folds over control) and the treated cv. ‘Tainung 57’ (1.86-folds over control). Under SWD conditions, there was a significant increase in total soluble sugar content in ‘Tainung 57’, by 3.91-, 2.39-, and 1.53-folds over well irrigated conditions when sprayed by 17, 34 and 51 μM PBZ, respectively (**Figure 2D**). In WD conditions, the enhancement in sugar classes followed a fructose > glucose > sucrose trend in the leaf tissues and this may play a key role in osmotic adjustment in sweet potato. Sucrose content (6.93 mg g^-1^ DW) in 34 μM PBZ pretreated plants grown under MWD was enriched by 6.08-folds over control. Glucose and fructose in cvs. ‘Japanese Yellow’ and ‘Tainung 57’ increased in relation to the degree of WD stress. Glucose and fructose contents peaked at 11.32 mg g^-1^ DW (2.87-folds over control) and 31.08 mg g^-1^ DW (4.21-folds over control) (**Table 3**). In SWD, sucrose, glucose, and fructose in positive check cv. ‘Japanese Yellow’ were enriched by 3.97-folds (3.02 mg g^-1^ DW), 1.99-folds (13.44 mg g^-1^ DW), and 5.19-folds (35.71 mg g^-1^ DW), respectively, over the control. In contrast, sucrose content in PBZ-pretreated plants cv. ‘Tainung 57’ in both well irrigated and SWD stress was unchanged (**Table 3**). However, glucose and fructose in the leaf tissues of 34 μM PBZ-pretreated plants of cv. ‘Tainung 57’ peaked at 13.35 mg g^-1^ DW (2.29-folds over control) and 26.94 mg g^-1^ DW (2.49-folds over control), respectively (**Table 3**).

**Table 3:** Sucrose, glucose and fructose content in leaf tissues of sweet potato pretreated with PBZ subsequently subjected to WW (47.5% SWC), mild water deficit (32.3% SWC; 7 days water withholding) or SWD (17.5% SWC; 14 days water withholding).

Treatment	Water deficit	7 days			14 days		
			
		Sucrose (mg g^-1^ DW)	Glucose (mg g^-1^ DW)	Fructose (mg g^-1^ DW)	Sucrose (mg g^-1^ DW)	Glucose (mg g^-1^ DW)	Fructose (mg g^-1^ DW)
**cv. ‘Japanese Yellow’ (+)**							
	WW	2.25bcd	3.25f	3.85e	0.76c	6.74d	6.88e
	WD	3.51b	9.12b	14.22c	3.02a	13.44a	35.71a
		(1.56)	(2.81)	(3.69)	(3.97)	(1.99)	(5.19)
**cv. ‘Tainung 57’ (-)**							
0 μM PBZ	WW	1.18d	5.72de	7.99d	1.82abc	5.34d	15.07d
	WD	2.32bcd	8.99b	16.30c	2.02ab	11.40ab	27.94b
		(1.97)	(1.57)	(2.04)	(1.11)	(2.14)	(1.85)
17 μM PBZ	WW	1.46cd	6.08de	8.00d	1.21bc	4.96d	4.44e
	WD	1.51cd	8.67bc	24.56b	1.70bc	11.47ab	28.28b
		(1.03)	(1.43)	(3.07)	(1.41)	(2.31)	(6.37)
34 μM PBZ	WW	1.14d	3.95ef	7.38d	1.01bc	5.84d	10.84d
	WD	6.93a	11.32a	31.08a	1.99ab	13.35a	26.94b
		(6.08)	(2.87)	(4.21)	(1.97)	(2.29)	(2.49)
51 μM PBZ	WW	1.44cd	6.83cd	15.95c	1.21bc	7.14cd	13.61d
	WD	2.92bc	13.25a	25.28b	1.60bc	12.79a	19.26c
		(2.03)	(1.94)	(1.59)	(1.32)	(1.79)	(1.42)


*Different letters in each column show significant difference at *p* ≤ 0.01 by Tukey’s HSD. Figures in parentheses represent sugar accumulation (folds) of water-deficit stressed plants compare to WW in each cultivar.*

**FIGURE 3:**
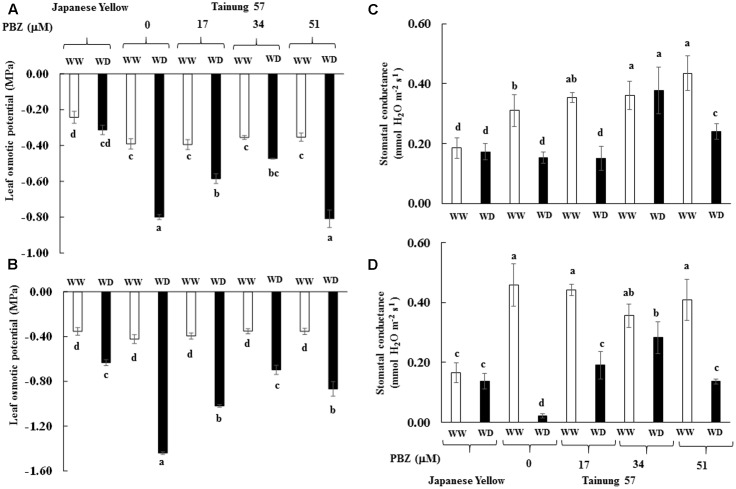
Leaf osmotic potential (Ψ_s_) and stomatal conductance (*g*_s_) of sweet potato pretreated with PBZ subsequently subjected to mild water deficit (32.3% SWC; 7 days water withholding) **(A,C)** or SWD (17.5% SWC; 14 days water withholding) **(B,D)**. Different letters in each bar show significant difference at *p* ≤ 0.01 by Tukey’s HSD. Data presented as mean ± SE.

Osmotic potential (Ψ_s_) in WD condition was decreased depending on the degree of stress. Leaf Ψ_s_ in 34 μM PBZ-pretreated plants of cv. “Tainung 57” was similar to the positive check (cv. “Japanese Yellow”) when subjected to MWD (**Figure 3A**). In SWD, leaf Ψ_s_ in all the treatments significantly declined (**Figure 3B**). It was confirmed that free proline and soluble sugar enrichment in the PBZ-pretreated sweet potato had a potential to control the osmotic potential in the leaf tissues, preventing the water loss *via* stomatal closure. In WD conditions, *g*_s_ in 34 μM PBZ-pretreated plants of cv. “Tainung 57” was stabilized in the same pattern as the positive check (**Figures 3C,D**).

### Photosynthetic Abilities

Photosynthetic pigments, i.e., Chl *a*, Chl *b*, and C_x+c_, in the positive check cv. ‘Japanese Yellow’ under WD conditions were maintained, whereas those in cv. ‘Tainung 57’ were significantly degraded, especially in SWD. Chl *a* and Chl *b* in WD stressed plants of cv. ‘Japanese Yellow’ were maintained (with only 3.84 and 3.65% degradation, respectively) under MWD and declined by 5.25 and 2.27%, respectively, under SWD. Total carotenoid (C_x+c_) content in leaf tissues of plants under SWD conditions significantly dropped by 14.32%, but was unaffected under MWD conditions (**Table 4**). In cv. ‘Tainung 57’, Chl *b* and C_x+c_ contents significantly declined by 8.30 and 27.41%, respectively, whereas Chl *a* was stable. Chl *a*, Chl *b*, and C_x+c_ in plants grown under SWD conditions were significantly reduced by 13.97, 6.64 and 34.63, respectively. The Chl *b* and C_x+c_ in 34 μM PBZ (5.32 and 7.98%) and 51 μM PBZ-pretreated plants (4.61 and 9.48%) were alleviated when subjected to MWD. In SWD, Chl *a* and Chl *b* in 34 μM PBZ (11.71 and 2.82%, respectively) and 51 μM PBZ-pretreated plants (9.59 and 4.90%, respectively) were maintained, whereas C_x+c_, a sensitive indicator, was significantly reduced (**Table 3**).

**Table 4:** Chlorophyll a (Chl *a*), chlorophyll b (Chl *b*), and total carotenoids (C_x+c_) content in leaf tissues of sweet potato pretreated with PBZ subsequently subjected to WW (47.5% SWC), mild water deficit (32.3% SWC; 7 days water withholding) or SWD (17.5% SWC; 14 days water withholding).

Treatment	Water deficit	7 days			14 days		
			
		Chl *a* (µg g^-1^ FW)	Chl *b* (µg g^-1^ FW)	C_x+c_ (µg g^-1^ FW)	Chl *a* (µg g^-1^ FW)	Chl *b* (µg g^-1^ FW)	C_x+c_ (µg g^-1^ FW)
**cv. ‘Japanese Yellow’ (+)**							
	WW	85.26ab	54.82a	7.25bc	88.94ab	53.66ab	8.03b
	WD	81.99b (3.84%)	52.80ab (3.65%)	6.83c (5.79%)	84.27ab (5.25%)	52.44ab (2.27%)	6.88c (14.32%)
**cv. ‘Tainung 57’ (-)**							
0 μM PBZ	WW	84.94ab	56.63a	8.50a	96.28a	55.29a	9.99ab
	WD	80.19b (5.59%)	51.93b (8.30%)	6.17c (27.41%)	82.83b (13.97%)	51.62b (6.64%)	6.53c (34.63%)
17 μM PBZ	WW	89.47a	58.16a	6.94c	92.91a	53.55ab	9.12ab
	WD	86.80ab (2.98%)	53.31ab (8.34%)	5.65d (18.59%)	81.16b (12.65%)	51.61b (3.62%)	6.06c (33.55%)
34 μM PBZ	WW	89.77a	56.21a	8.27ab	98.89a	56.09a	10.16a
	WD	81.49b (9.23%)	53.22ab (5.32%)	7.61bc (7.98%)	85.54ab (11.71%)	54.51ab (2.82%)	7.60bc (25.20%)
51 μM PBZ	WW	93.12a	53.78ab	8.12ab	95.69a	53.43ab	10.31a
	WD	85.76ab (7.90%)	51.30b (4.61%)	7.35bc (9.48%)	86.51ab (9.59%)	50.83b (4.90%)	7.20bc (30.17%)


*Different letters in each column show significant difference at *p* ≤ 0.01 by Tukey’s HSD. Figures in parentheses represent pigment degradation percentage of water-deficit stressed plants compare to WW in each cultivar.*

**FIGURE 4:**
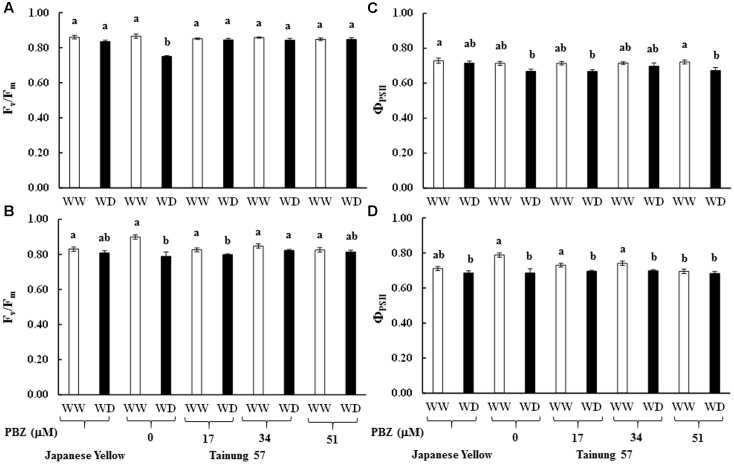
Maximum quantum yield of PSII (*F*_v_/*F*_m_) and proton yield of PSII (Φ_PSII_) of sweet potato pretreated with PBZ subsequently subjected to mild water deficit (32.3% SWC; 7 days water withholding) **(A,C)** or SWD (17.5% SWC; 14 days water withholding) **(B,D)**. Different letters in each bar show significant difference at *p* ≤ 0.01 by Tukey’s HSD. Data presented as mean ± SE.

**FIGURE 5:**
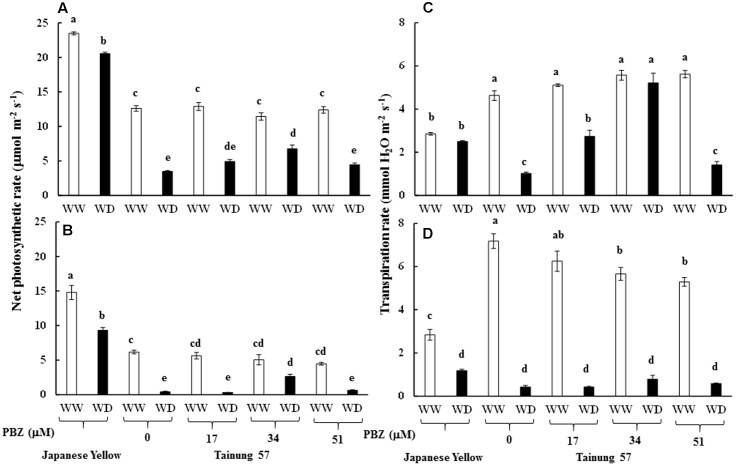
Net photosynthetic rate (*P*_n_) and transpiration rate (*E*) of sweet potato pretreated with PBZ subsequently subjected to mild water deficit (32.3% SWC; 7 days water withholding) **(A,C)** or SWD (17.5% SWC; 14 days water withholding) **(B,D)**. Different letters in each bar show significant difference at *p* ≤ 0.01 by Tukey’s HSD. Data presented as mean ± SE.

**FIGURE 6:**
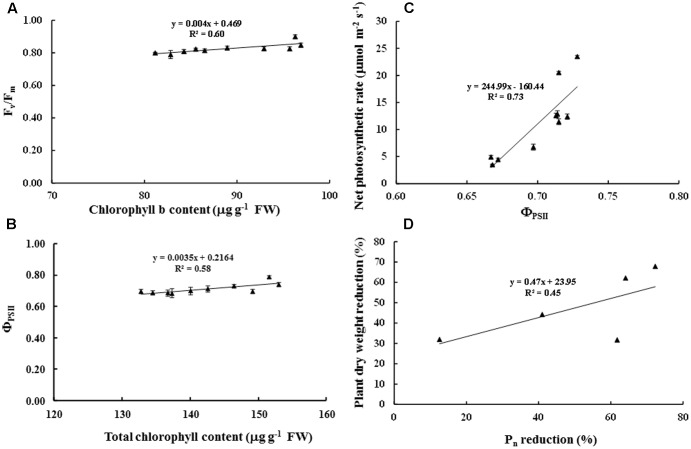
Relationship between chlorophyll a content and *F*_v_/*F*_m_ **(A)**, total chlorophyll content and Φ_PSII_ **(B)**, Φ_PSII_ and net photosynthetic rate (*P*_n_) **(C)**, and *P*_n_ reduction and plant dry weight reduction of sweet potato pretreated with PBZ subsequently subjected to water deficit conditions **(D)**. Data presented as mean ± SE.

Maximum quantum yield of PSII (*F*_v_/*F*_m_) in cv. ‘Tainung 57’ grown under MWD was significantly dropped (13.40% diminution), whereas it was maintained in cv. ‘Japanese Yellow’ (only 2.01% diminution) (**Figure 4A**). In MWD, *F*_v_/*F*_m_ in PBZ-pretreated plants was retained. Similarly, *F*_v_/*F*_m_ in 34 and 51 μM PBZ-pretreated plants grown under SWD was maintained (only 2.95 and 1.34% diminution, respectively) (**Figure 4B**). Photon yield of PSII (Φ_PSII_) in MWD was maintained, whereas it was declined by 6.8% in 51 mM PBZ-pretreated plants (**Figure 4C**). In contrast, Φ_PSII_ in 51 mM PBZ-pretreated plants was maintained (1.58% diminution) similar to cv. ‘Japanese Yellow’. A significant diminution of Φ_PSII_ (13.08% reduction) was revealed in cv. ‘Tainung 57’ plants without PBZ-pretreatment (**Figure 4D**). *P*_n_ in cv. ‘Tainung 57’ was lower than that of cv. ‘Japanese Yellow’ and significantly dropped when plants exposed to MWD and SWD (**Figures 5A,B**). In MWD, the *P*_n_ in cv. ‘Japanese Yellow’ was maintained (12.6% reduction), whereas it was decreased by 72.3% in cv. ‘Tainung 57’ plants without PBZ-pretreatment (**Figure 5A**). Moreover, *P*_n_ in cv. ‘Japanese Yellow’ grown under SWD was reduced by 37.09% and it was sharply declined by 93.36% in cv. ‘Tainung 57’ non-treated plants. Interestingly, *P*_n_ in 34 mM PBZ-pretreated plants under MWD and SWD was alleviated (with only 41 and 47.5% reduction, respectively) when compared with other treatments (**Figures 5A,B**). In addition, transpiration rate (*E*) in cv. ‘Japanese Yellow’ and cv. ‘Tainung 57’ pretreated with 34 mM PBZ subsequently grown under MWD was sustained (with only 6.46% reduction) (**Figure 5C**). In SWD, *E* was a sensitive parameter that has significantly declined (**Figure 5D**). A positive correlation between Chl *a* content and *F*_v_/*F*_m_ (*R*^2^ = 0.60), TC content and Φ_PSII_ (*R*^2^ = 0.58), Φ_PSII_ and *P*_n_ (*R*^2^ = 0.73), *P*_n_ reduction and plant dry weight (*R*^2^ = 0.45) was demonstrated (**Figure 6**).

## Discussion

Storage root yield of sweet potato was improved by 37 μM PBZ pretreatment, especially in the water shortage at the early growth stage. Drought stress delay the time of flowering in potted red firespike PBZ pretreatment (0.24 mg plant^-1^ soil drench) improved it and maintained the number of flowers (6 flowers plant^-1^) equal to control (Rezazadeh et al., 2016). In other studies, fruit yield of tomato (*Lycopersicon esculentum* Mill. cv. Hybrid Alex 63) pretreated with 50 mg L^-1^ PBZ was 1.37-folds higher than non-treated plants. This yield declined by 4.79% when the pretreated plants were subjected to WD conditions at 60% field capacity (FC) (Mohamed et al., 2011). In addition, tomato fruit yield (3.89 kg plant^-1^) and number of fruits (31 fruits plant^-1^) in 30 mg L^-1^ PBZ pretreated tomato plants were maintained when exposed to drought condition (Latimer, 1992). Overall, there were enhanced yield performances, in PBZ-pretreated plants than non-pretreated plants under both WW and WD conditions (**Table 1**).

Root fresh- and dry weight traits in sweet potato were identified as very sensitive to the drought stress, which was improved by PBZ-pretreatment in the present study. Studies on English oak, European beech, and Lombardy poplar tree species also indicate a 4.56-, 3.50-, and 3.67-fold increase in root dry weight, respectively, when treated with 0.5 g PBZ plant^-1^ (Percival and Sacre, 2014). In WD stress (water withholding for 3 weeks), root dry weight of horse chestnut was recovered (with only 18.4% reduction) by 500 mg L^-1^ PBZ treatment (Percival and Noviss, 2008). Shoot biomass (mg plant^-1^) in irrigated finger millets with or without 100 μM PBZ was unchanged. Shoot dry weight (37.17% reduction) and root dry weight (13.04% reduction) of 60 mg L^-1^ PBZ-treated tomato grown under drought condition were maintained (Latimer, 1992). Moreover, dry weight of 50 mg L^-1^ PBZ pretreated plants declined by 20.45% as compared to 36.77% reduction in non-treated plants (Bayat and Sepehri, 2012). Shoot dry weight in turf grass, *Festuca arundinacea* L. cv. Master and *Lolium perenne* L. cv. Barrage, was very sensitive to the WD conditions (25% FC), resulting in a 95.45 and 97.68% decrease, respectively, whereas this was improved by using 30 mg L^-1^ PBZ treated seeds (with up to 3.14% reduction) (Shahrokhi et al., 2011).

Paclobutrazol, an anti-GA, has been reported as plant growth retardant. Plant height, a good indicator of PBZ function, in *Phillyrea angustifolia* was reduced depending on the degree of PBZ concentration in the soil (Fernández et al., 2006). In Pathumma (*Curcuma alismatifolia* Gagnep. cv. Chiang Mai Pink), shoot height of 3.75 g L^-1^ PBZ-treated plants was inhibited by 48.93% compared to the untreated plants. In addition, when these plants were treated with 1,500 mg L^-1^ PBZ (soil drenching) under water withholding conditions for 20 days (5% SWC) and 30 days (4% SWC), the shoot length was maintained when compared to non-treated plants (Jungklang et al., 2016). Likewise, 2.0 mg pot^-1^ PBZ soil drenching resulted in reduced shoot height in sunflower and zinnia by 26.3 and 42.1%, respectively (Ahmad et al., 2014). *Syzygium myrtifolium* (Roxb.) Walp. treated with 3.75 g L^-1^ PBZ had decreased plant height and leaf area by 19.93 and 60.02%, respectively (Roseli et al., 2012). Leaf area of 30 mL L^-1^ PBZ-pretreated *P. angustifolia* plants grown under WW conditions was reduced by 83.25%. Therefore, the growth performances of PBZ-pretreated plants were improved when exposed to MWD conditions (I60) and then declined under SWD conditions (I40) (Fernández et al., 2006). Correspondingly, shoot height, root length, and leaf area of 10 mg L^-1^ PBZ-pretreated peanut plants were improved compared to the plants without PBZ treatment when exposed to drought conditions (Sankar et al., 2014a).

Free proline enrichment in sweet potato cv. ‘Japanese Yellow’ under WD conditions has been well established as a WD tolerance indicator (Yooyongwech et al., 2013). In the WD condition, free proline biosynthesis via pyrroline-5-carboxylate synthetase (P5CS) and pyrroline-5-carboxylate reductase (P5CR) was upregulated to promote proline as major osmoprotectant at the cellular level (Delauney and Verma, 1993; Kishor et al., 2005). In two cultivars of sweet potato (“Untacip” and “Huambachero”), free proline was regulated by WD conditions in both leaf and storage root tissues (Rodríguez-Delfin et al., 2012). In cell suspension culture, free proline in sweet potato cv. ‘Tainung 57’ grown under 0.6 M sorbitol-induced WD conditions was enriched by 5.25-folds over control (Wang et al., 1999). In the present study, free proline content in the leaf tissues of sweet potato cv. ‘Tainung 57’ under MWD conditions were unchanged. In contrast, free proline content in PBZ-pretreated plants under SWD conditions was significantly increased when compared with cv. ‘Japanese Yellow’ without PBZ. This is the first report of proline regulation by a combination of PBZ and water deficiency in sweet potato. It is possible that PBZ regulates the free proline accumulation, especially in combination with WD stress (Hajihashemi and Ehsanpour, 2013). Similarly, free proline content in 50 mg L^-1^ PBZ treated tomato plants grown under 60% FC peaked at 54.56 mg g^-1^ FW (1.52-fold over control) (Mohamed et al., 2011). In contrast, free proline content in 10 mg L^-1^ PBZ-pretreated peanut under WD conditions (1.04-folds over control) was lower than non-treated plants (1.49-folds over control) (Sankar et al., 2014b).

Total soluble sugar enrichment in sweet potato cell culture under 0.6 M sorbitol-induced water deficiency has been well established (Wang et al., 1999). It may function as major osmoprotectant at the cellular level when plants were exposed to WD conditions, leading to control the osmotic potential in the leaf and stomata closure to prevent the water loss (Sánchez et al., 1998; Iannucci et al., 2002). Recent studies indicate that glucose and fructose content in WD stressed sweet potato cultivars “‘Tainung 57”’ and “PROC65-3” increased, whereas sucrose content was unaffected (Yooyongwech et al., 2016). It was confirmed that the activity of acid soluble invertase enzyme was reached threefolds over control to convert sucrose into fructose and glucose when plants were subjected to WD stress (Pelleschi et al., 1997). In addition, fructose, glucose, sucrose, and total soluble sugar in English oak, Lombardy poplar, and European beach treated by 0.5 g PBZ plant^-1^ soil drench application increased by 1.3- to 2.0-folds over control (Percival and Sacre, 2014). In untreated and PBZ treated (50 mg L^-1^) WD stressed tomato (60% FC), there was a 1.16- and 1.52-fold increase in total soluble sugars (Mohamed et al., 2011). In 6% PEG-induced WD stress, water soluble carbohydrate and reducing sugars were significantly declined in untreated *S. rebaudiana* Bertoni, whereas the sugar content was elevated by 2 mg L^-1^ upon PBZ foliar application (Hajihashemi and Ehsanpour, 2013). Total soluble sugar enrichment in PBZ-pretreated sweet potato may play a vital role in the osmotic adjustment at cellular level of plant under WD conditions. In PBZ-pretreated plants, sugar, a major soluble carbohydrate derived from transitory starch degradation, was evidently observed (Hare et al., 1998; Gupta and Kaur, 2005) that maintains the leaf water potential under drought conditions (Zhu et al., 2004). In strawberry tree, water consumption was reduced by 10% (60 mg PBZ plant^-1^) and 20% (100 mg PBZ plant^-1^) compared to the control, causing lower *g*_s_ and CO_2_ assimilation after treatment with PBZ (Navarro et al., 2007). It seems to help the plants in acclimatization before the exposure to WD conditions (Cha-um et al., 2009). Under drought conditions, PBZ elevates the *g*_s_ and water use efficiency in WW conditions as validated in potted red firespike (Rezazadeh et al., 2016), tomato (Pal et al., 2016), and wheat (Dwivedi et al., 2017). It is possible that PBZ improves the stomatal function to prevent the water loss from transpiration when exposed to drought stress (Berova and Zlatev, 2003).

Recent studies indicate that, photosynthetic pigments in sweet potato cultivars, ‘PROC 65-3,’ ‘Japanese Yellow,’ and “Tainung 57” under low SWC were degraded, in relation to the degree of WD stress (Yooyongwech et al., 2014). Similarly, TC in sweet potato cv. Beauregard was reduced depending on the decrease in soil moisture content, while it was stable in cv. Evangeline (Gajanayake et al., 2014). In tomato, TC content in the leaf tissues of 50 mg L^-1^ PBZ-pretreated plants under 60% FC was maintained (Mohamed et al., 2011). In horse chestnut, TC and C_x+c_ in plants under WD stress was declined by 69.39 and 65.61%, respectively, and then improved to 55.03 and 55.63%, respectively, on 500 mg L^-1^ PBZ treatment (Percival and Noviss, 2008). In addition, the improvement of TC and C_x+c_ in 1-2 mg L^-1^ PBZ pretreated plants was confirmed in *S. rebaudiana* grown under 6% polyethylene glycol (PEG)-induced drought condition (Hajihashemi and Ehsanpour, 2013).

Overall photosynthetic abilities, i.e., chlorophyll fluorescence, *P*_n_ and *E*, in PBZ-pretreated sweet potato were improved than those without PBZ. *F*_v_/*F*_m_ and *P*_n_ in horse chestnut plants under WD conditions declined by 58.02 and 62.95%, respectively, and was recovered by 33.33 and 57.01%, respectively, in the presence of 500 mg L^-1^ PBZ (Percival and Noviss, 2008). Apart from these parameters, PBZ has been studied to show positive effects on cuticular wax biosynthesis (Jenks et al., 2001), leaf water potential (Zhu et al., 2004; Bañon et al., 2009), water content (Jungklang and Saengnil, 2012; Hajihashemi and Ehsanpour, 2013), and water consumption (Navarro et al., 2007). Hence, PBZ has a cumulative effect in enhancing WD tolerance in plant species (Gilley and Fletcher, 1997; Berova and Zlatev, 2003; Navarro et al., 2009). In present study, a positive relation in physiological (photosynthetic abilities) and growth (plant dry weight) parameters was demonstrated (**Figure 6**). Similarly, TC concentration in the leaf tissues of *Quercus robur* and *Q. ilex* treated with 2 and 4 ppm PBZ foliar spray alleviates in relation to maintain chlorophyll fluorescence (performance index or PI) and *P*_n_, leading to improve survival percentage (Percival and Albalushi, 2007).

## Conclusion

Total soluble sugar and free proline in 34 μM PBZ pretreated sweet potato cv. ‘Tainung 57’ may play a critical role in the osmotic adjustment to stabilize photosynthetic pigments, enhance photosynthetic abilities and control water transpiration, leading to sustained growth and storage root yield against WD stress.

## Author Contributions

This study was designed, directed and coordinated by SC and SY as principal investigator, provided conceptual and technical guidance for all aspects. CT, RT, and TS performed and analyzed the biochemical, physiological, morphological, and yield traits.

## Conflict of Interest Statement

The authors declare that the research was conducted in the absence of any commercial or financial relationships that could be construed as a potential conflict of interest.
