# Supplementary material for: Water-Deficit Tolerance in Sweet Potato [Ipomoea batatas (L.) Lam.] by Foliar Application of Paclobutrazol: Role of Soluble Sugar and Free Proline
Source: Front Plant Sci. 2017 Aug 8;8:1400. doi: 10.3389/fpls.2017.01400 (PMC5550687; doi:10.3389/fpls.2017.01400)
Supplement: Supplementary file 1 [file Image_1.PDF]

Supplementary

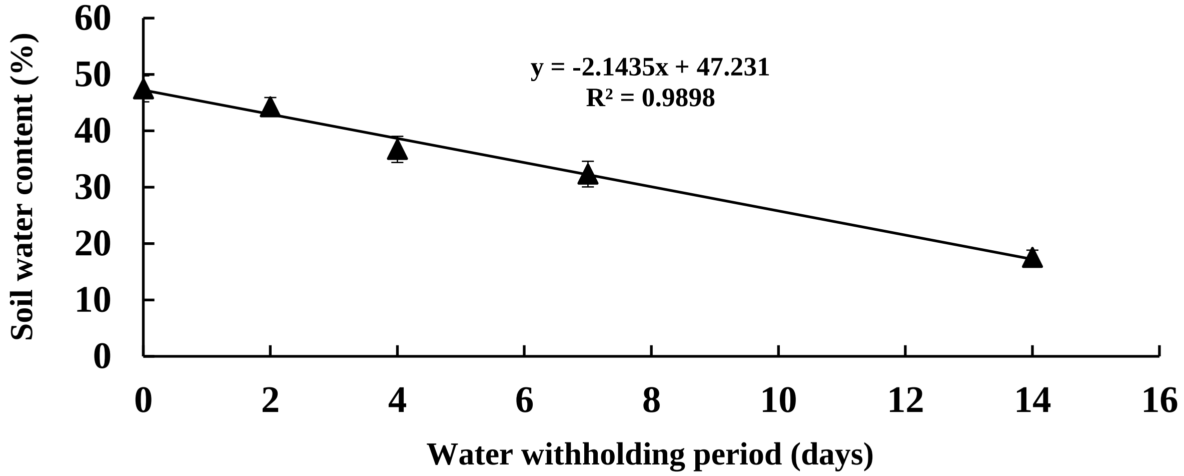

Fig. S1 Soil water content in the soil after water withholding period.

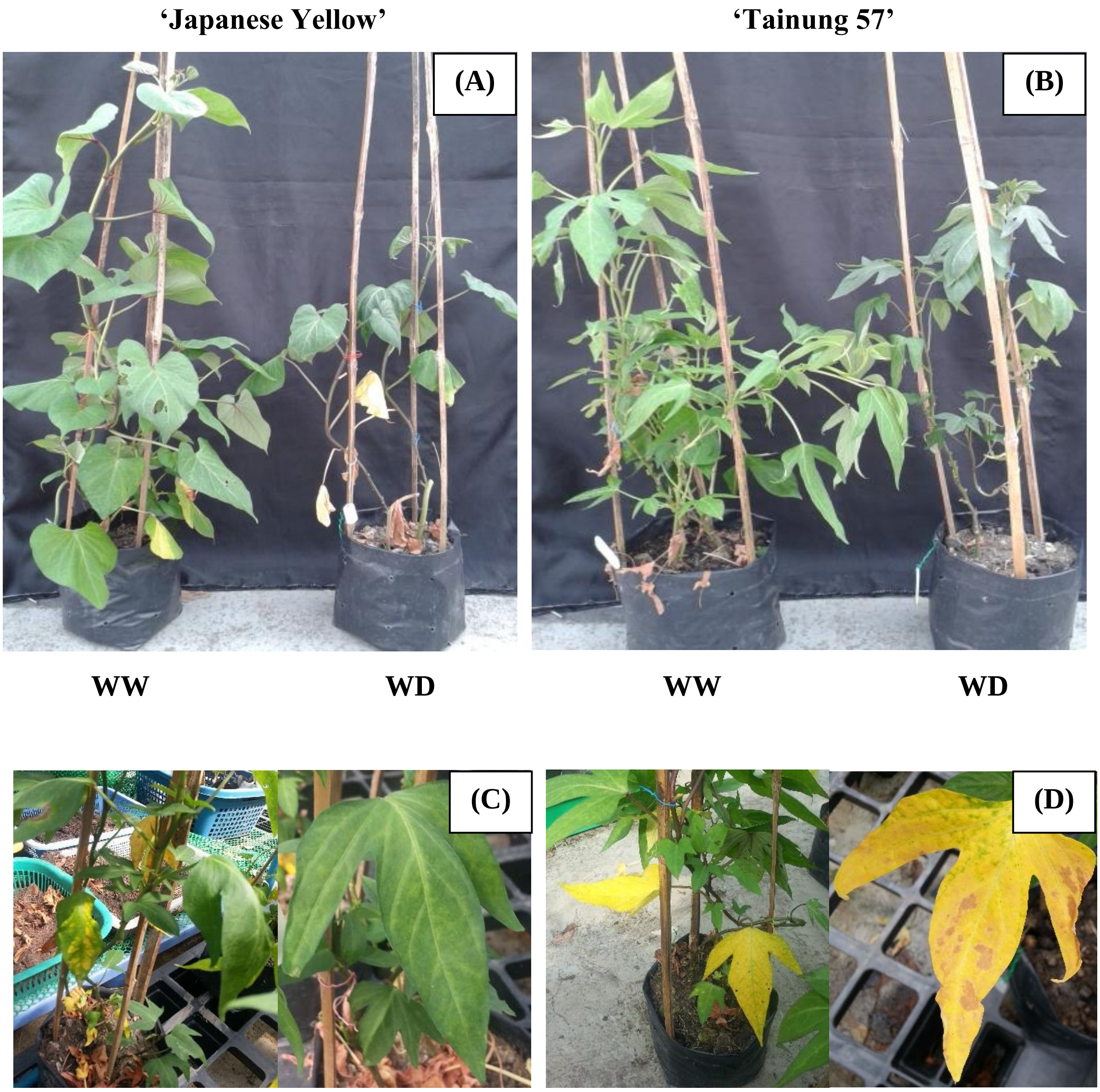

Fig. S2 Plant morphological characteristics of sweet potato cvs. 'Japanese Yellow' (A; positive check) and "Tainung 57" (B; 34 μM PBZ pretreatment) grown under well watering (WW) and water deficit condition (WD). Leaf wilting (C) and leaf chlorosis (D) symptoms in sweet potato cv. 'Tainung 57' without PBZ-pretreated plants grown under water deficit stress (17.5% SWC) were revealed.

Table S1 Significant level in two-way analysis of variance on vine length (VL), leaf length (LL), leaf width (LW), number of leaves (NL), leaf osmotic potential (Ψ_s), free proline (Pro), sucrose (Suc), glucose (Gluc), fructose (Fruc), maximum quantum yield of PSII (F_v/F_m), photon yield of PSII (Φ_{PSII}), net photosynthetic rate (P_n), stomatal conductance (g_s) and transpiration rate (E) in sweet potato PBZ-pretreated plants grown under moderate water deficit stress.

Treatment	VL	LL	LW	NL	Ψ_s	Pro	Suc	Gluc	Fruc
PBZ	NS	**	**	*	**	**	**	**	**
WD	NS	*	*	NS	**	**	**	**	**
PBZ × WD	**	NS	NS	NS	**	*	**	**	**

Treatment	Chl_a	Chl_b	C_{x+c}	F_v/F_m	Φ_{PSII}	P_n	g_s	E
PBZ	*	*	**	NS	NS	**	**	**
WD	**	**	**	*	*	**	**	**
PBZ × WD	*	**	**	*	*	**	**	**

Table S2 Significant level in two-way analysis of variance on vine length (VL), leaf length (LL), leaf width (LW), number of leaves (NL), leaf osmotic potential (Ψ_s), free proline (Pro), sucrose (Suc), glucose (Gluc), fructose (Fruc), maximum quantum yield of PSII (F_v/F_m), photon yield of PSII (Φ_{PSII}), net photosynthetic rate (P_n), stomatal conductance (g_s) and transpiration rate (E) in sweet potato PBZ-pretreated plants grown under severe water deficit stress.

Treatment	VL	LL	LW	NL	Ψ_s	Pro	Suc	Gluc	Fruc
PBZ	NS	NS	**	**	**	**	**	**	**
WD	**	**	**	**	**	**	**	**	**
PBZ × WD	**	NS	NS	NS	**	**	**	**	**

Treatment	Chl_a	Chl_b	C_{x+c}	F_v/F_m	Φ_{PSII}	P_n	g_s	E
PBZ	*	*	**	NS	NS	**	**	**
WD	**	**	**	*	*	**	**	**
PBZ × WD	*	*	**	*	*	*	**	**

Table S3 Significant level in two-way analysis of variance on storage root yield, vine fresh weight, root fresh weight, vine dry weight and root dry weight in sweet potato PBZ-pretreated plants exposed to severe water deficit subsequently recovery prior to harvesting stage.

**Treatment**	**Storage root yield**	**Vine FW**	**Root FW**	**Vine DW**	**Root DW**
PBZ	**	**	**	**	**
WD	**	**	**	**	**
PBZ × WD	**	**	**	**	NS
